# Human Papillomavirus Knowledge and Communication Skills: A Role-Play Activity for Providers

**DOI:** 10.15766/mep_2374-8265.11150

**Published:** 2021-04-23

**Authors:** Theresa M. Fiorito, Leonard R. Krilov, Jeannine Nonaillada

**Affiliations:** 1 Attending Physician, Department of Pediatrics, Division of Pediatric Infectious Diseases, NYU Langone Long Island Hospital; Assistant Professor, Department of Pediatrics, NYU Long Island School of Medicine; 2 Chief, Department of Pediatrics, Division of Pediatric Infectious Diseases, NYU Langone Long Island Hospital; Professor, Department of Pediatrics, NYU Long Island School of Medicine; Chair, Department of Pediatrics, NYU Langone Long Island Hospital; 3 Associate Professor, Department of Medicine, Division of Geriatric Medicine, NYU Long Island School of Medicine; Assistant Dean, Faculty Development and Mentoring, NYU Long Island School of Medicine

**Keywords:** Human Papillomavirus (HPV), Alphapapillomavirus, Vaccines, Role-Play, Communication Skills, Pediatric Infectious Diseases, Pediatrics

## Abstract

**Introduction:**

Human papillomavirus (HPV) infection and related cancers are a major cause of morbidity and mortality worldwide. Routine vaccination against HPV is recommended for patients starting at age 9–12 years. Discussing this vaccine with parents of young children can be challenging for clinicians. Barriers include parental beliefs, strength and quality of clinician recommendations, physician knowledge of HPV disease and vaccines, and provider comfort levels with discussing sexuality.

**Methods:**

Our interactive workshop began with a predidactic role-play session addressing common concerns about the HPV vaccine where participants took turns playing a concerned parent or provider. We then gave a 30-minute didactic lecture and conducted a postdidactic role-play session to practice communication skills in promoting the HPV vaccine. All participants completed pre- and postintervention knowledge and skill self-assessments.

**Results:**

Twenty-eight pediatric residents and medical students participated. We observed significant improvement in their ability to appropriately recommend the HPV vaccine in the postdidactic role-play (all *p*s < .02). Learner knowledge improved from pre- to postintervention (from 34% to 100%, *p* < .0025, based on average score), as did self-perceived comfort and confidence levels (from 3.6 to 4.3, *p* < .0001, average score based on a 5-point Likert scale).

**Discussion:**

An interactive workshop utilizing role-play supplemented by a didactic lecture was effective in improving participants’ knowledge, communication skills, comfort levels, and confidence levels regarding HPV disease and vaccines. The workshop offers a practical and interpersonal approach to improving learners’ skills in discussing the HPV vaccine with parents.

## Educational Objectives

By the end of this session, learners will be able to:

1. Make an effective recommendation of the human papillomavirus (HPV) vaccine to parents, using strategies proven to increase vaccine uptake.
2. Cite risks and statistics regarding HPV disease.
3. Identify components and adverse effects of the HPV vaccine.
4. Implement recommended strategies to effectively address parental vaccine hesitancy regarding HPV.

## Introduction

Human papillomavirus (HPV) is the most common sexually transmitted infection in the United States.^1^ The American Academy of Pediatrics and the Advisory Committee on Immunization Practices of the Centers for Disease Control and Prevention recommend routine HPV vaccination for females and males starting at age 9–12 years.^1,2^ Despite this recommendation, rates of HPV vaccination remain low nationwide.^3^ There are many parental barriers to vaccination, including a low perceived risk of HPV infection, lack of necessity prior to sexual debut, potential side effects, and concerns that the vaccine will encourage younger children to engage in sexual activity.^4–8^ Deficiencies in strength and quality of clinician recommendations, physician knowledge of the vaccine, and provider comfort levels with discussing sexuality are described in the literature.^8,9^ Some studies characterize a gender bias among providers, with lower rates of HPV vaccination among males.^10,11^

Multiple studies address potential strategies to increase acceptance of the HPV vaccine. Decision-making can be influenced by parental satisfaction with provider communication about HPV vaccine, specifically the strength of the provider's recommendation.^9,12,13^ Barriers to vaccination may conceivably be overcome with educational activities that allow providers an opportunity to improve both knowledge and communication skills.

Studies on effective education on HPV among providers are ongoing. Several *MedEdPORTAL* publications have shared successful examples of HPV education, including flipped-classroom modules utilizing a combination of quizzes, clinical application exercises, and lectures for first-year medical students^14^ and case-based approaches.^15^ While many educational activities about HPV recommend certain practices, such as strong clinician recommendations, they lack content on how specifically to change these practices. Education should focus on practical application of interpersonal approaches, which is what makes role-play such an appealing tool for the present resource.^16^

Across the literature in medical education, role-play is used as a training tool to improve communication skills among providers. To allow trainees to practice communication skills in palliative care, one study from Seattle Children's Hospital implemented role-play wherein senior residents played the patient's parent. Postsession surveys demonstrated a significant improvement in residents’ confidence in their communication skills and in comprehension of the family experience.^17^ A workshop performed at the University of North Carolina at Chapel Hill with fourth-year medical students and primary care providers used role-play to increase skills, knowledge, and confidence regarding advance care planning. Following the workshop, attitudes and knowledge significantly improved.^18^

To our knowledge, this is the first workshop using role-play in the HPV vaccine education of pediatric providers. We evaluated the effectiveness of the workshop in increasing learner knowledge and communication skills about HPV, self-reported comfort levels discussing sexuality, and self-reported confidence levels promoting the HPV vaccine with parents.

## Methods

The NYU Langone Long Island Hospital Institutional Review Board reviewed this project. The project met criteria for exempt research (reference no. 1315859-1, date of approval: September 26, 2018).

### Theoretical Framework

**Figure 1.**
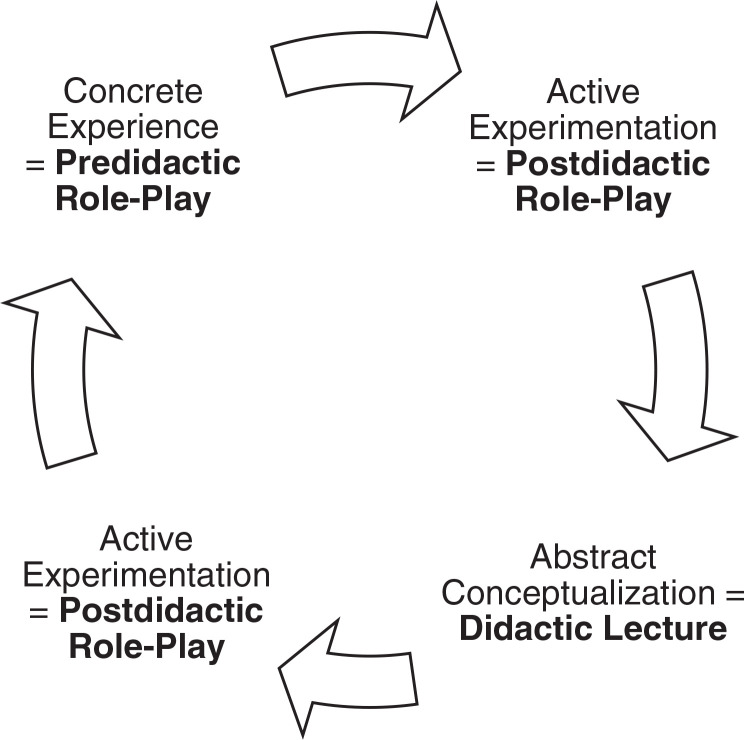
Kolb's experiential theory as applied to the design of our role-play workshop.

We implemented adult learning theory by providing an environment in which mistakes were expected, so that learners would not feel threatened or uncomfortable (predidactic role-play session). The didactic session and postdidactic role-play provided a basis for continued learning. At the beginning of the workshop, we stated a clear goal of increasing HPV acceptance among parents and patients, with the end goal of individual self-advancement. The learning journey involved active participation, and the role-play was designed to be problem oriented.^19^ Kolb's experimental theory was applied to the design of the workshop, as detailed in Figure 1.^20^ We designed the predidactic role-play as the concrete experience, with the postdidactic role-play corresponding to active experimentation.

### Workshop Sign-Up and Schedule

We scheduled this workshop as part of our hospital's educational lecture series for pediatric residents and medical students doing a pediatric clerkship. We did not require any prereading. The ideal group size for us was approximately 25–30 participants based on the size of the room we had available, as more participants would have likely caused distractions and increased noise. Facilitators were provided with practical instructions for running the workshop (Appendix A). The workshop consisted of a pre- and postintervention knowledge and skill self-assessment (Appendix B), a predidactic role-play session (Appendix C), a didactic lecture (Appendix D), and a postdidactic role-play session (Appendix C again), for a total time of 140 minutes. We held a debriefing session after each role-play. (For a more detailed workshop schedule, see Appendix A.)

### Workshop Room Setup

We set up a large space with pairs of chairs facing each other and six feet apart per group of two. If this setup is not feasible in future implementations, multiple rooms may need to be used to minimize distractions. We used clipboards for the scripts; if clipboards are not available, facilitators could consider small tables for each group to serve as recording surfaces. We set up a projector with a working computer in the front of the room for the slide presentation.

### Role-Play

We developed case questions for the role-play based on educational activities currently available online.^21–23^ The role-play was designed with eight questions and lasted 30 minutes. The same script was used for both the pre- and postdidactic role-play sessions. The first role-play took place before the lecture to challenge participants to generate failures and to drive and motivate them to learn and reflect.^24^ Paired participants alternated playing a provider or concerned parent of an 11-year-old girl (15 minutes in each role, four questions for each provider). The parent received a script with questions to ask the provider (Appendix C) and recorded the provider's answers. After the didactic lecture, the role-play exercise was repeated (postdidactic role-play). We provided a rubric (Appendix F) with appropriate answers to each question so that pre- and postdidactic results could be quantified and compared directly to each other.

### Debriefing

After each 30-minute role-play, the facilitator could ask questions of the learners, as noted in Appendix A, in a debriefing that lasted 10–15 minutes. These questions were designed to extract feedback regarding what learners thought had gone well and what could be improved for future sessions. The postdidactic role-play focused on feedback as well as strategies employed by learners to answer questions.

### Didactic Session

We gave a 20-minute PowerPoint lecture on HPV disease and vaccines (Appendix D) after the predidactic role-play. We also discussed strategies for answering difficult questions posed in the role-play exercise.

### Program Assessment

Before and after the workshop, we distributed a knowledge and skill self-assessment (Appendix B, with answer key in Appendix E). We developed and piloted the assessment to reflect the contents of the didactic component. Communication skills were assessed by comparing responses recorded by the parent in the pre- and postdidactic role-play exercises. We also constructed a postparticipation evaluation of this activity to strengthen the findings of future workshops (Appendix G).

## Results

**Figure 2.**
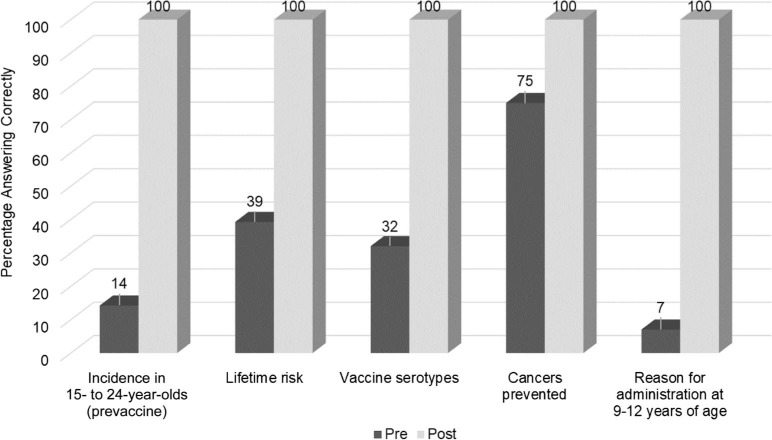
Comparison of pre- and postintervention answers to questions assessing knowledge of human papillomavirus disease and vaccines (*N* = 28). Answers were assessed as a binary response; a correct answer was defined as achieving 1 point, and an incorrect answer was defined as achieving <1 point. All pre-post differences were statistically significant (*p* < .0001).

All available pediatric residents (*n* = 25), as well as medical students (*n* = 3) doing their pediatric clerkship, completed the workshop (*N* = 28). Residents on night shifts or vacation did not attend. Figure 2 compares the percentages of participants with correct responses on the pre- and postintervention knowledge and skill self-assessments. Overall, the participants achieved a significant increase in total learner knowledge, with 100% of them answering the knowledge questions correctly following the workshop. The largest deficit in knowledge preintervention was “Reason for administration at 9–12 years of age.” The majority of participants focused only on early vaccination prior to sexual activity. Just two participants described enhanced immunogenicity (requiring two vs. three doses) with HPV vaccination prior to age 16.

Participants self-rated their comfort and confidence on 5-point Likert scales (1 = *not at all comfortable/confident,* 5 = *extremely comfortable/confident*). We observed a significant increase in comfort level discussing sexuality (from mean score 3.5 preworkshop to 4.2 postworkshop, *p* < .0001) and in confidence in promoting the HPV vaccine following the activity (from mean score 3.7 preworkshop to 4.5 postworkshop, *p* < .0001).

**Figure 3.**
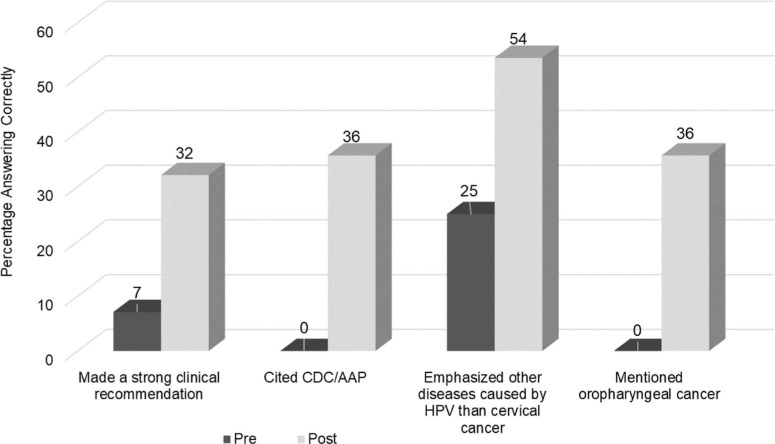
Comparison of human papillomavirus (HPV) vaccination opening recommendations in pre- versus postdidactic role-plays (*N* = 28). All pre-post differences were statistically significant (*p* < .02). Abbreviations: AAP, American Academy of Pediatrics; CDC, Centers for Disease Control and Prevention.

A paired analysis of pre- versus postintervention HPV vaccination opening recommendations yielded significant improvement in the postdidactic role-play (Figure 3). The remaining responses were recorded solely for observational purposes because, in the pilot study, role-play questions differed between pre- and postdidactic sessions. This has been corrected for future studies so that a direct comparison can be made.

## Discussion

We developed a workshop utilizing a combination of role-play, discussion, and didactics to address HPV vaccine hesitancy. To address potential gaps in provider knowledge of HPV, we acquainted learners with common parental concerns in the predidactic role-play and provided a preworkshop assessment. The predidactic role-play also served as a starting point for reflective observations.^24^ The debriefing sessions allowed for feedback and review of content. The didactic lecture provided learners with both factual data and strategies proven to increase HPV uptake. The postdidactic role-play allowed learners to hone their communication skills.

To our knowledge, this is the first resource utilizing role-play in HPV provider education. Our results suggest that the design and structure of this workshop were effective in improving participants’ knowledge, communication skills, confidence levels, and comfort levels regarding HPV disease and vaccines. It is possible that the interactive approach kept learners actively engaged in the role-plays, which proved advantageous for retaining knowledge, as evidenced by the difference in assessment scores pre- and postworkshop.^25^ While giving the didactic lecture, we noticed that a number of learners were startled by some of the statistics and facts about HPV. The social aspect of the workshop promoted active learning, while the immediate opportunity to practice furthered skill development.^26^ The use of Kolb's experiential theory may have contributed to this workshop's success, as participants were able to immediately apply their ideas and concepts to practice in the postdidactic role-play.^20^

Dividing the group into pairs complemented adult learning theory, as learners might otherwise have felt intimidated by a large group and being put on the spot. We noticed that participants were eager to convey any knowledge acquired from the workshop, citing the lifetime risk of HPV, epidemiological statistics, and so on when making recommendations. The volume of information that participants were able to absorb impressed us; this level of enthusiasm may reflect the degree of effectiveness of the workshop.

The workshop had several limitations. A small sample size may have led to an overestimation of the workshop's effectiveness. We would have liked to separate results based on educational levels, particularly medical student scores compared to resident scores; however, our small sample size did not allow for this comparison. Knowledge and self-confidence might decline over time, so booster sessions could longitudinally assess how well study participants retain their newly acquired knowledge. Standardized patients were not used, but many studies using them have shown favorable responses from participants.^27–29^

In the pilot study, participants playing the provider were not given a script. We have augmented this here so that the participants playing the provider can contextualize the case for themselves and better answer the questions. However, only the participants playing the parent should be given the script with the grading rubric on it. Also in the pilot study, questions differed between the pre- and postdidactic role-plays (with the exception of the initial pitch of the HPV vaccine), which prevented direct comparison of role-play responses. For future studies, matching questions in the role-play will provide a direct comparison of effectiveness; we have corrected this in the role-play scripts published here.

In reflecting on the design and delivery of this educational intervention, we feel that having to record responses during the role-play may have been distracting for participants. For future sessions, prespecified objectives for each role-play question have been developed: Instead of recording responses, participants can simply check off boxes. In other role-play questions, appropriate answers are provided so that the pre- and postdidactic results can be quantified and compared directly (Appendix F).

This role-play workshop provides a practical and interpersonal approach that allows participants to immediately apply newly acquired knowledge and skill sets. Evidence of increased knowledge, communication skills, comfort discussing sexuality, and confidence in promoting the HPV vaccine was identified following the didactic session and in the postdidactic role-play. Future studies will focus on longitudinal retention of learner knowledge and the impact of these improved skills on HPV vaccination rates among our pediatric practices.

## Appendices

Facilitator Instructions.docxPre- and Postworkshop Self-Assessment.docxRole-Play Script.docxHPV Didactic Lecture.pptxSelf-Assessment Answer Key.docxRole-Play Rubric.docxPostparticipation Evaluation.docx
All appendices are peer reviewed as integral parts of the Original Publication.
