# Supplementary material for: Human Papillomavirus Knowledge and Communication Skills: A Role-Play Activity for Providers
Source: MedEdPORTAL. 2021 Apr 23;17:11150. doi: 10.15766/mep_2374-8265.11150 (PMC8063629; doi:10.15766/mep_2374-8265.11150)
Supplement: Supplementary file 1 — Facilitator Instructions.docxPre- and Postworkshop Self-Assessment.docxRole-Play Script.docxHPV Didactic Lecture.pptxSelf-Assessment Answer Key.docxRole-Play Rubric.docxPostparticipation Evaluation.docx [file mep_2374-8265.11150-s001.zip › C. Role-Play Script.docx]

**Role Play Script: can give to “Provider”**

Scenario: **Parent** is at **provider**’s office with her 11-year old daughter for a well-check.

1. **Parent**: “Hello. I am here with my 11-year old daughter for a well-check appointment. I would like to know what vaccines are due for my child today?” (Prompt provider to give their “best pitch” of the HPV vaccine.)
2. **Parent**: “How do you get this disease?”
3. **Parent**: “What are the chances she’ll get this disease in her lifetime?”
4. **Parent**: “My daughter is not having sex yet! I’ll wait until she is older.”

**NOW SWITCH ROLES!!!**

1. **Parent**: “This vaccine is only going to encourage my daughter to have sex!”
2. **Parent**: “I’m sorry, but this vaccine really just seems too new for me.”
3. **Parent**: “Well, what are the side effects of this vaccine?”
4. **Parent**: “This sounds good. Can I get this vaccine for myself, too??” (Note: Parent is 35 years old.)
