# Supplementary material for: Human Papillomavirus Knowledge and Communication Skills: A Role-Play Activity for Providers
Source: MedEdPORTAL. 2021 Apr 23;17:11150. doi: 10.15766/mep_2374-8265.11150 (PMC8063629; doi:10.15766/mep_2374-8265.11150)
Supplement: Supplementary file 1 — Facilitator Instructions.docxPre- and Postworkshop Self-Assessment.docxRole-Play Script.docxHPV Didactic Lecture.pptxSelf-Assessment Answer Key.docxRole-Play Rubric.docxPostparticipation Evaluation.docx [file mep_2374-8265.11150-s001.zip › D. HPV Didactic Lecture.pptx]

## Slide 1
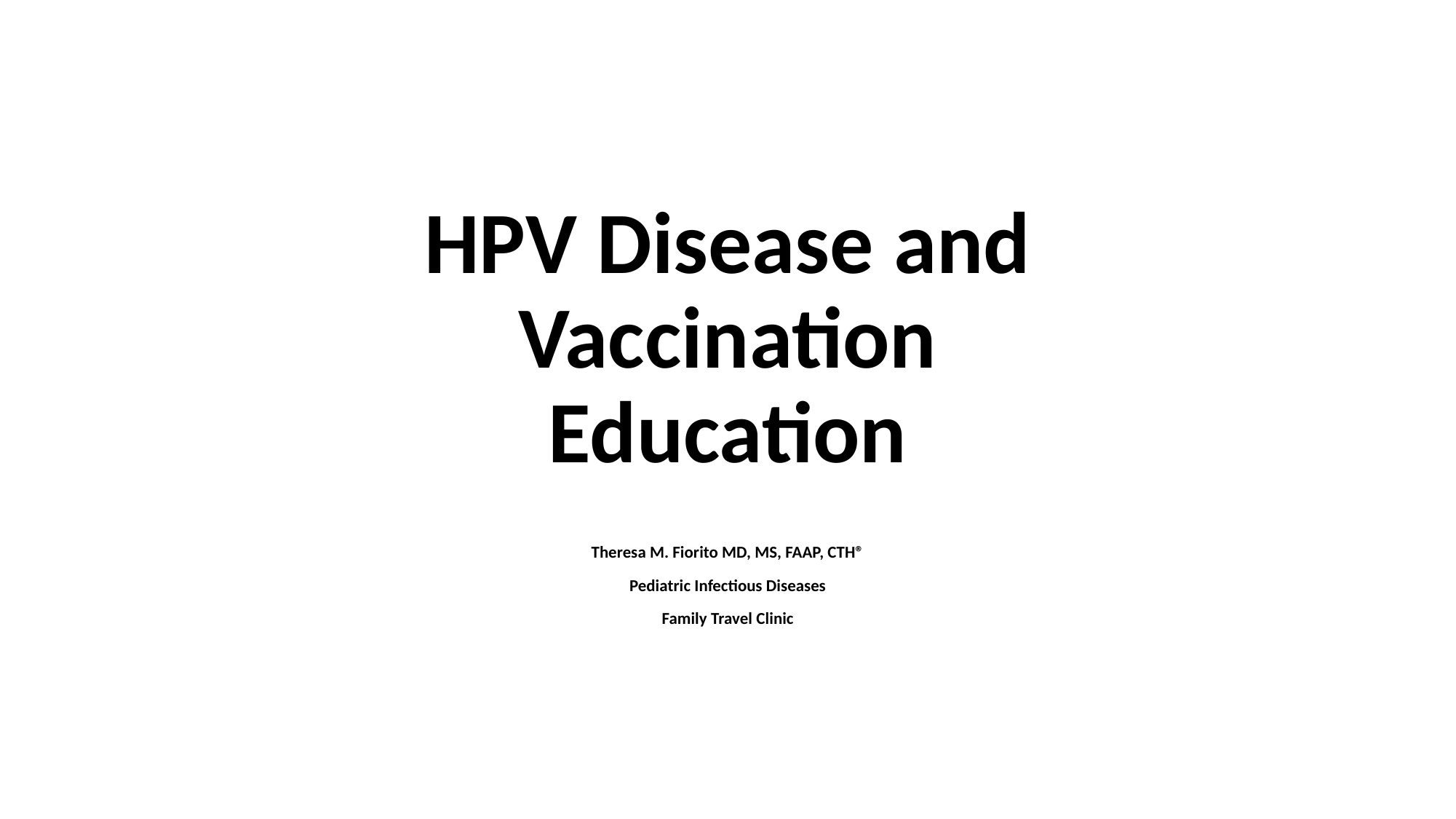

# HPV Disease and Vaccination Education
Theresa M. Fiorito MD, MS, FAAP, CTH®
Pediatric Infectious Diseases
Family Travel Clinic

## Slide 2
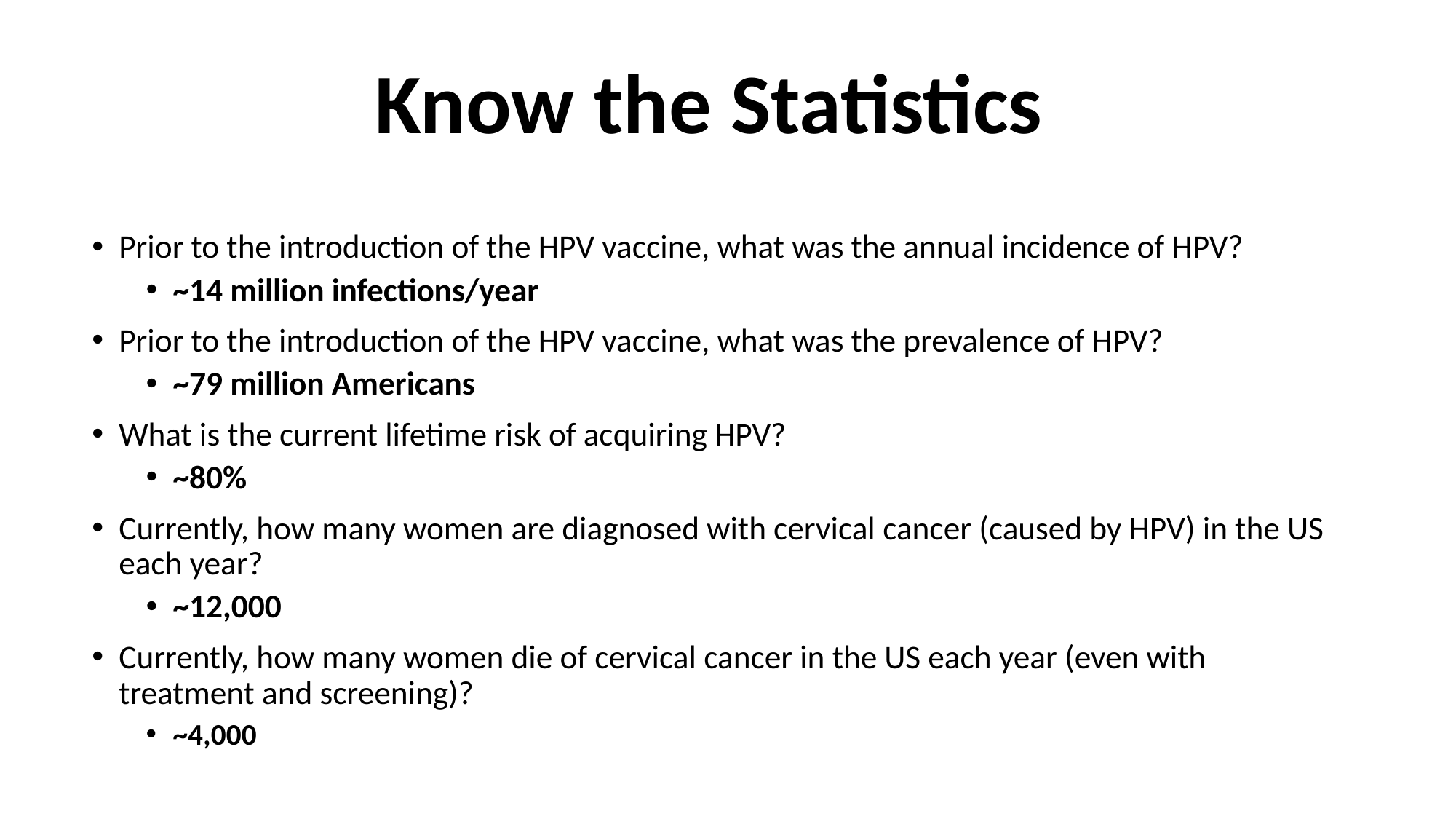

# Know the Statistics
Prior to the introduction of the HPV vaccine, what was the annual incidence of HPV?
~14 million infections/year
Prior to the introduction of the HPV vaccine, what was the prevalence of HPV?
~79 million Americans
What is the current lifetime risk of acquiring HPV?
~80%
Currently, how many women are diagnosed with cervical cancer (caused by HPV) in the US each year?
~12,000
Currently, how many women die of cervical cancer in the US each year (even with treatment and screening)?
~4,000

## Slide 3
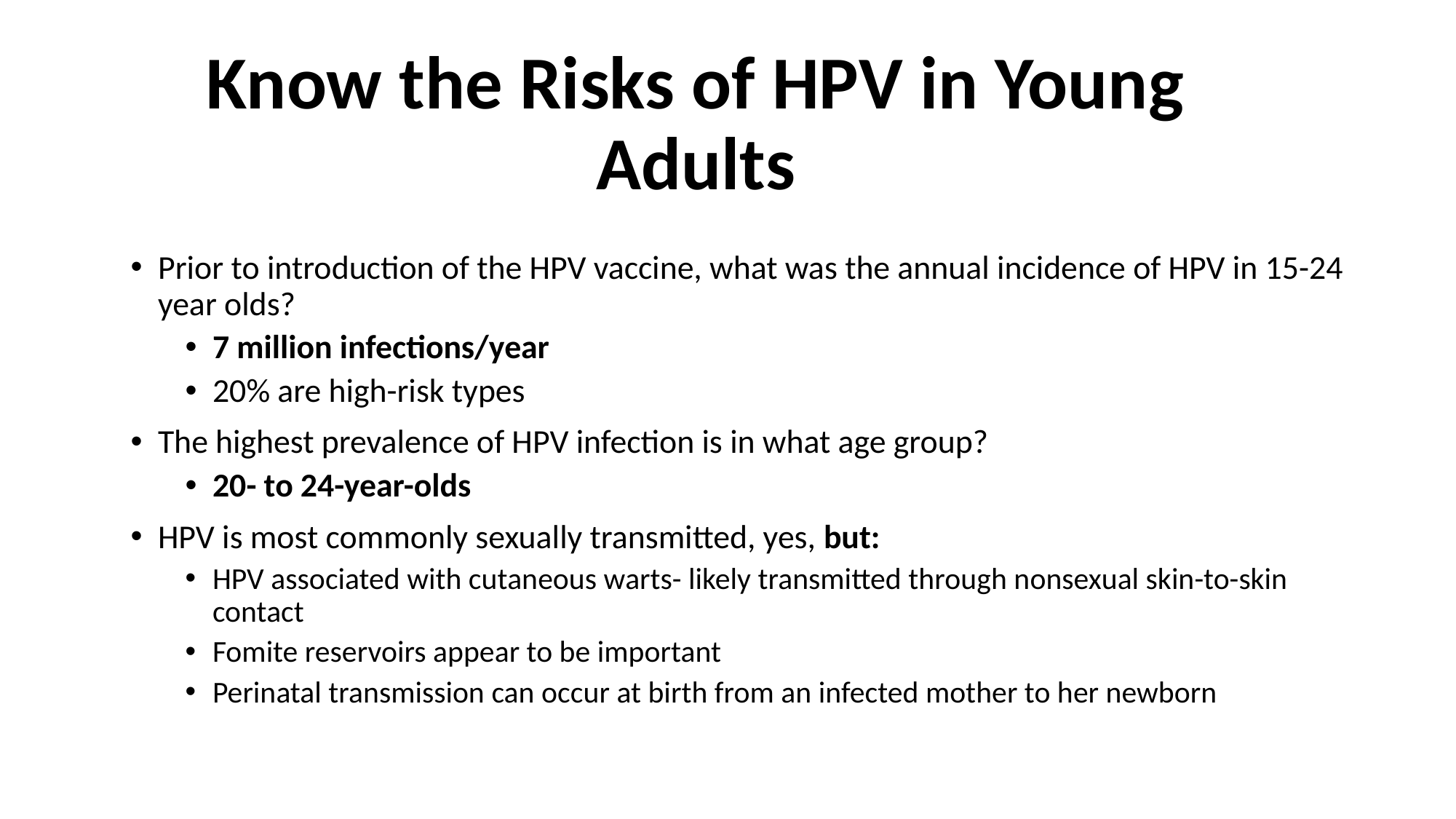

# Know the Risks of HPV in Young Adults
Prior to introduction of the HPV vaccine, what was the annual incidence of HPV in 15-24 year olds?
7 million infections/year
20% are high-risk types
The highest prevalence of HPV infection is in what age group?
20- to 24-year-olds
HPV is most commonly sexually transmitted, yes, but:
HPV associated with cutaneous warts- likely transmitted through nonsexual skin-to-skin contact
Fomite reservoirs appear to be important
Perinatal transmission can occur at birth from an infected mother to her newborn

## Slide 4
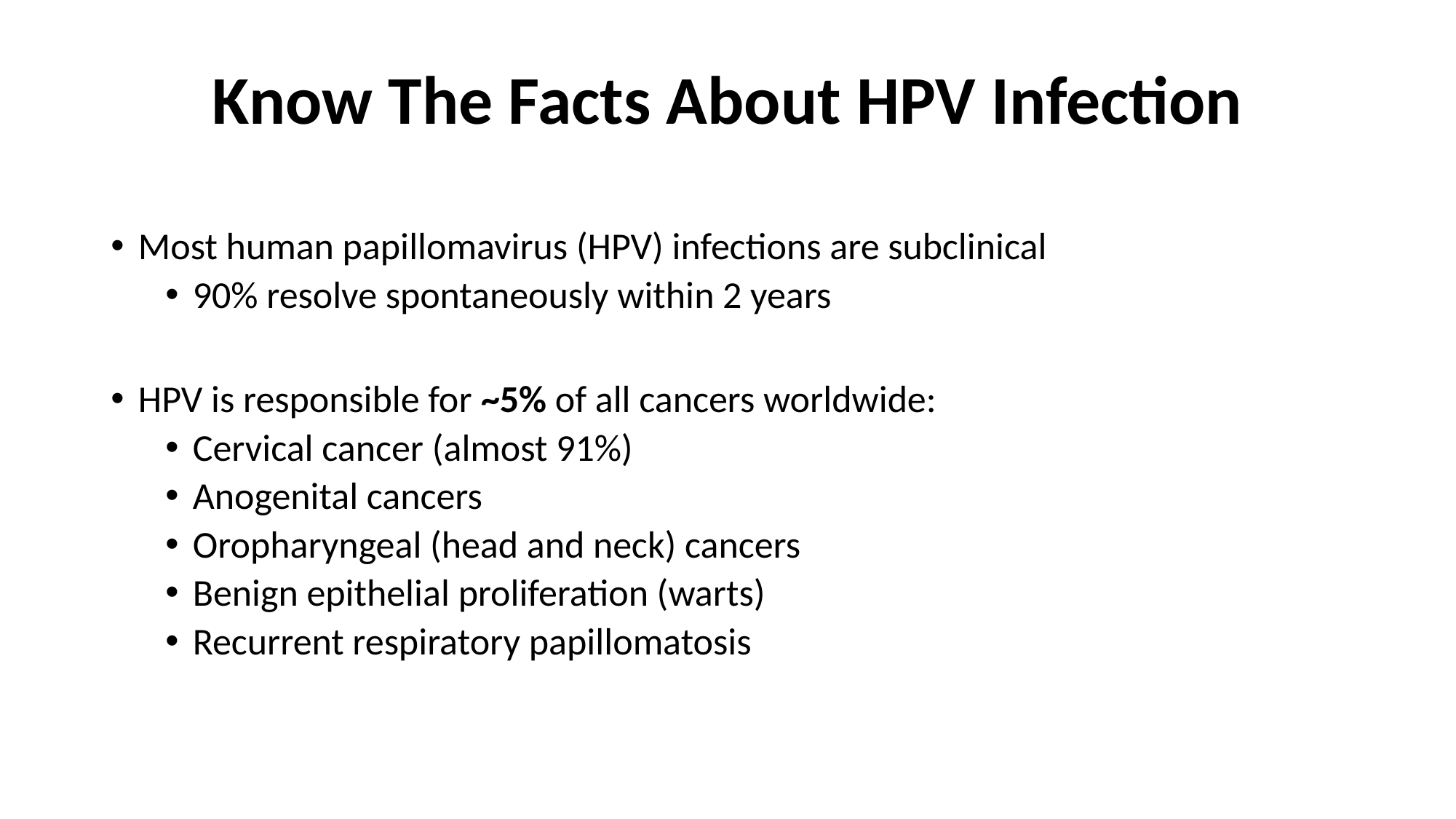

# Know The Facts About HPV Infection
Most human papillomavirus (HPV) infections are subclinical
90% resolve spontaneously within 2 years
HPV is responsible for ~5% of all cancers worldwide:
Cervical cancer (almost 91%)
Anogenital cancers
Oropharyngeal (head and neck) cancers
Benign epithelial proliferation (warts)
Recurrent respiratory papillomatosis

## Slide 5
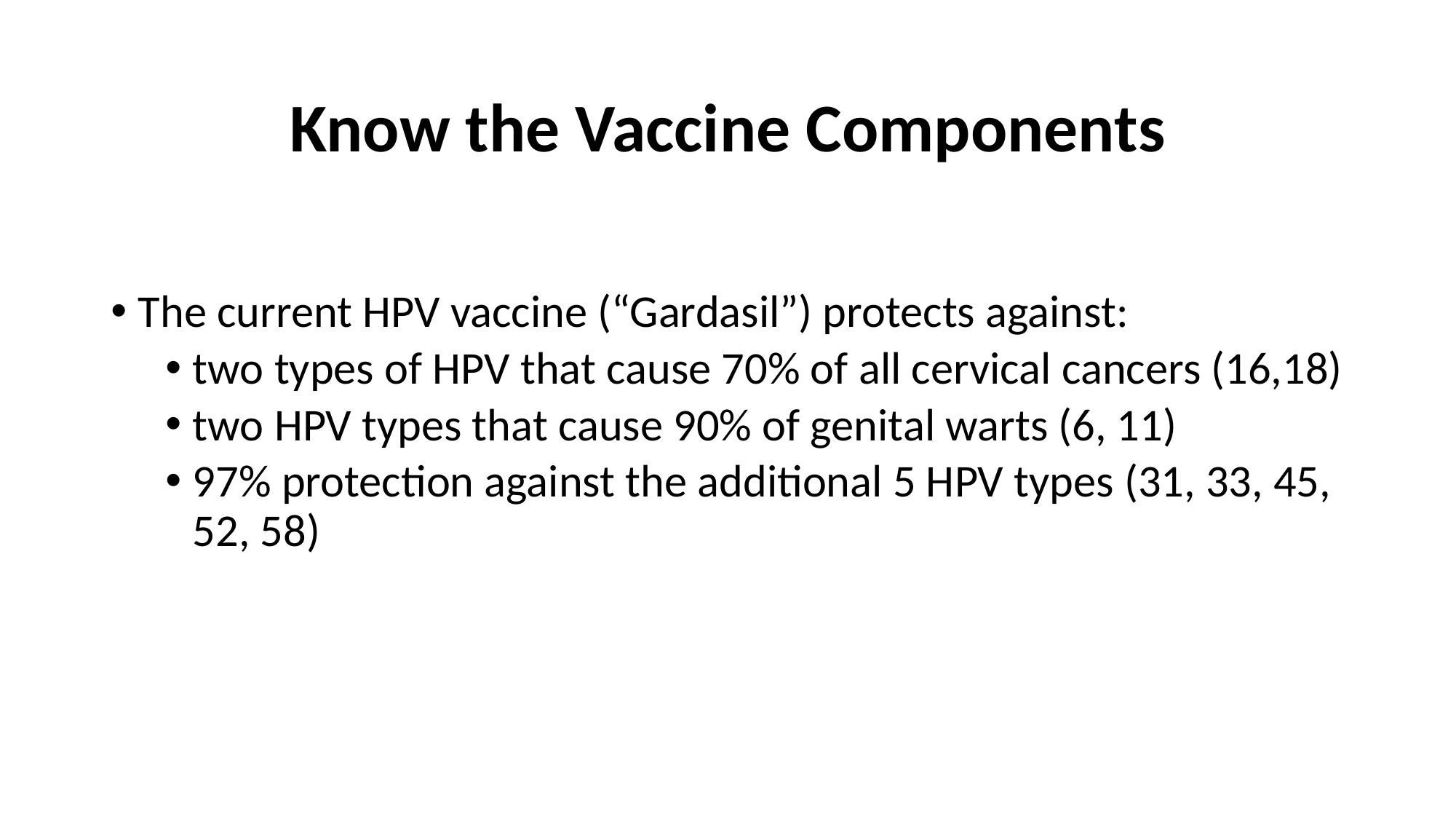

# Know the Vaccine Components
The current HPV vaccine (“Gardasil”) protects against:
two types of HPV that cause 70% of all cervical cancers (16,18)
two HPV types that cause 90% of genital warts (6, 11)
97% protection against the additional 5 HPV types (31, 33, 45, 52, 58)

## Slide 6
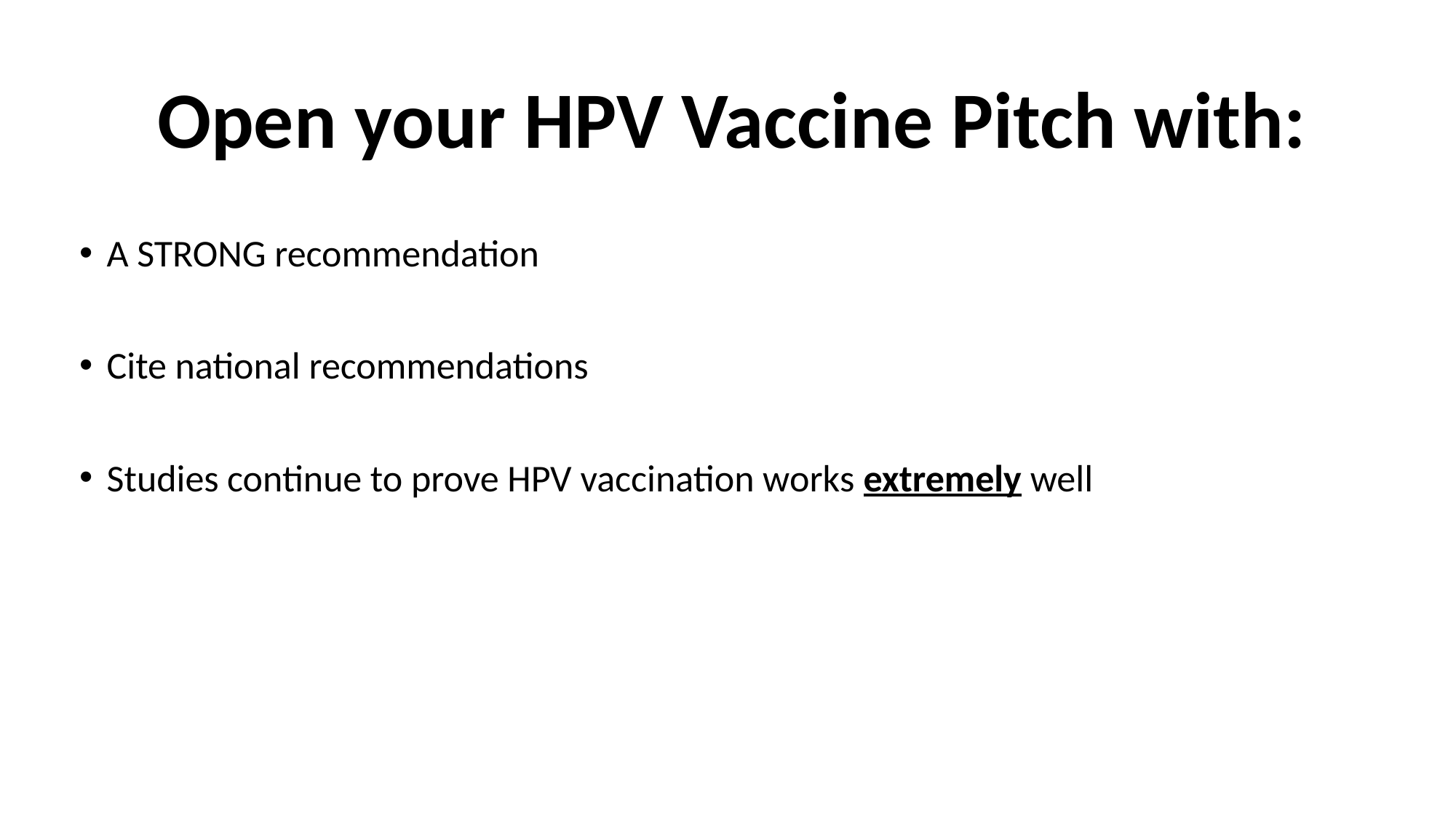

# Open your HPV Vaccine Pitch with:
A STRONG recommendation
Cite national recommendations
Studies continue to prove HPV vaccination works extremely well

## Slide 7
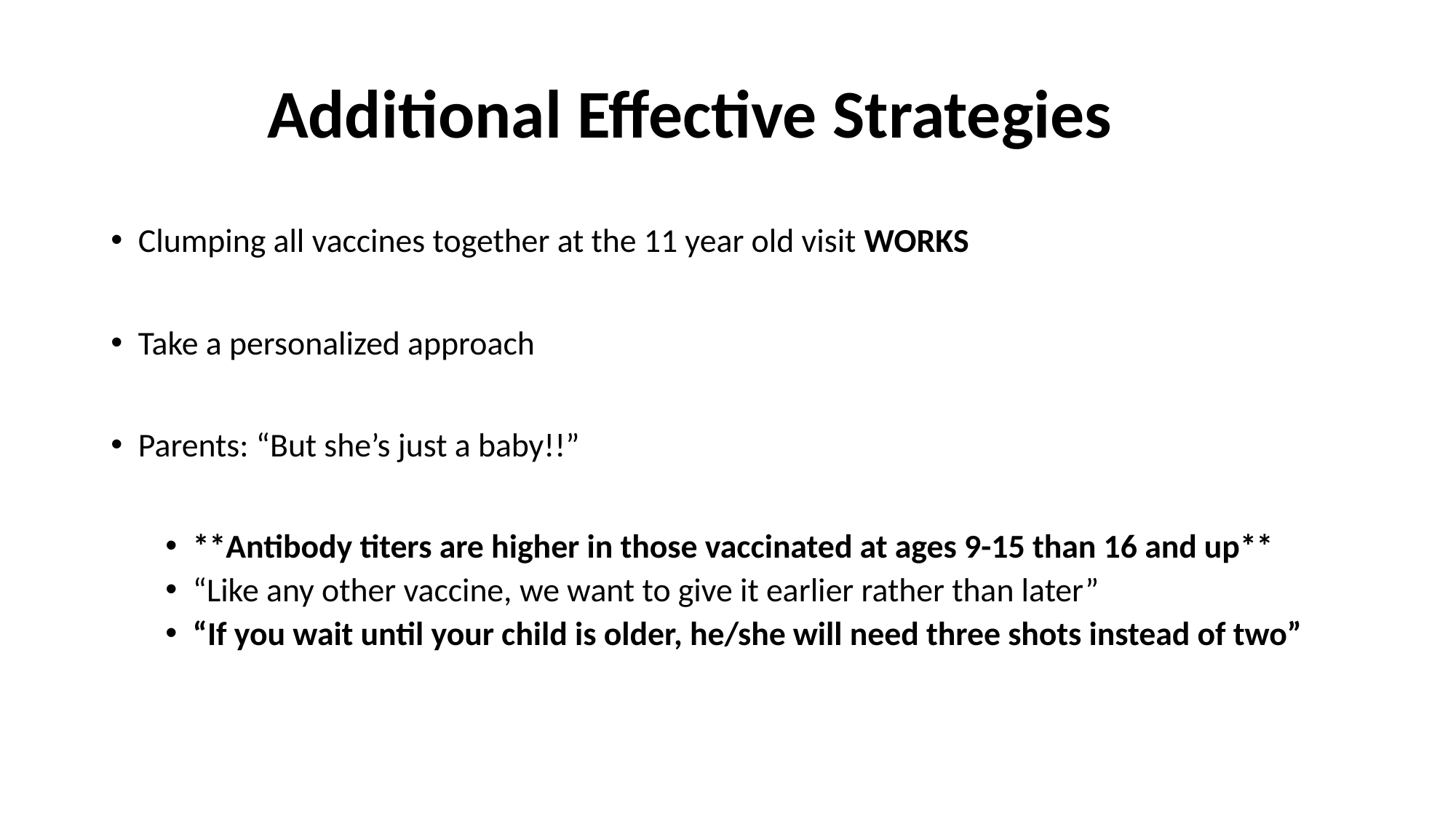

# Additional Effective Strategies
Clumping all vaccines together at the 11 year old visit WORKS
Take a personalized approach
Parents: “But she’s just a baby!!”
**Antibody titers are higher in those vaccinated at ages 9-15 than 16 and up**
“Like any other vaccine, we want to give it earlier rather than later”
“If you wait until your child is older, he/she will need three shots instead of two”

## Slide 8
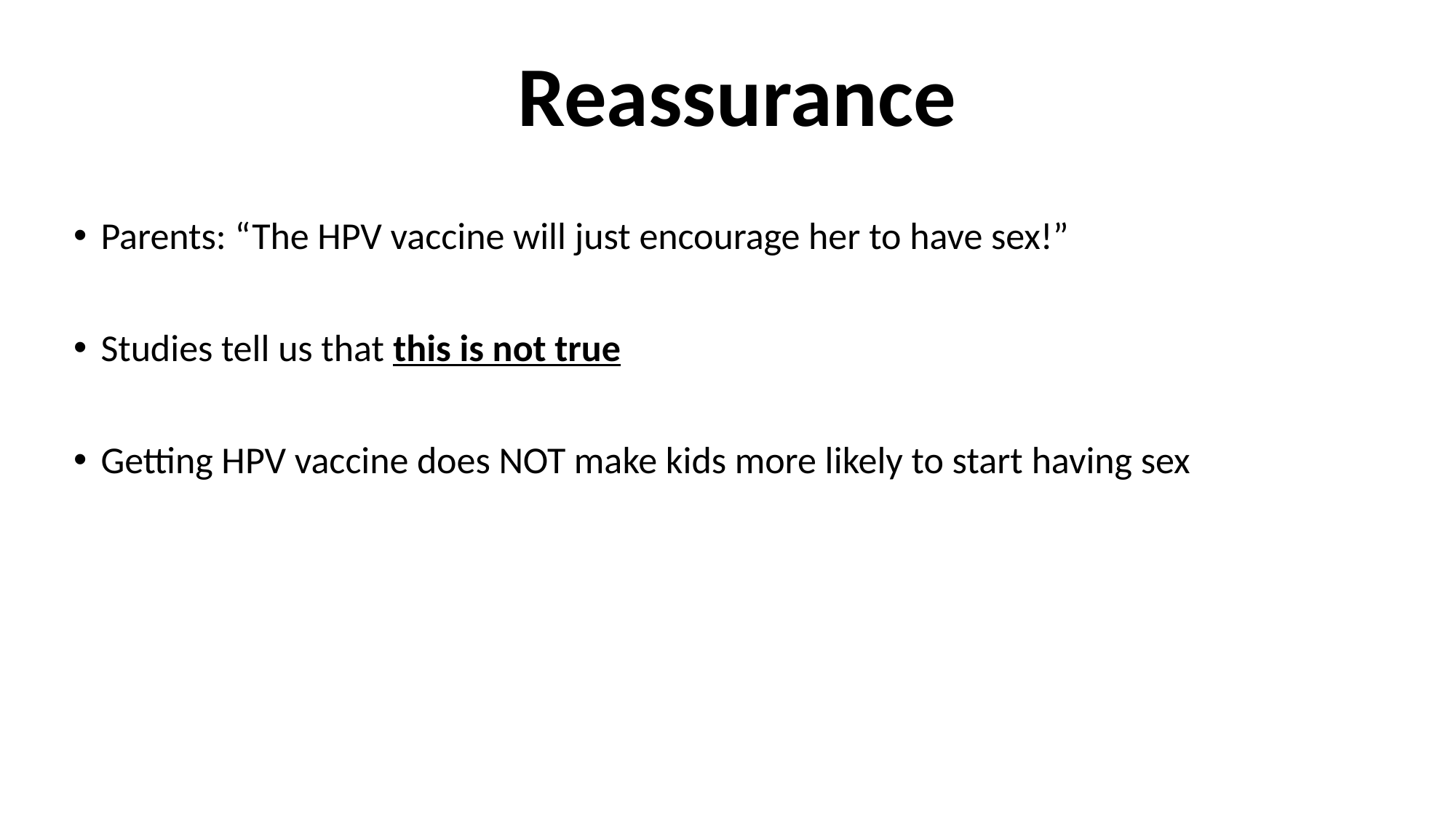

# Reassurance
Parents: “The HPV vaccine will just encourage her to have sex!”
Studies tell us that this is not true
Getting HPV vaccine does NOT make kids more likely to start having sex

## Slide 9
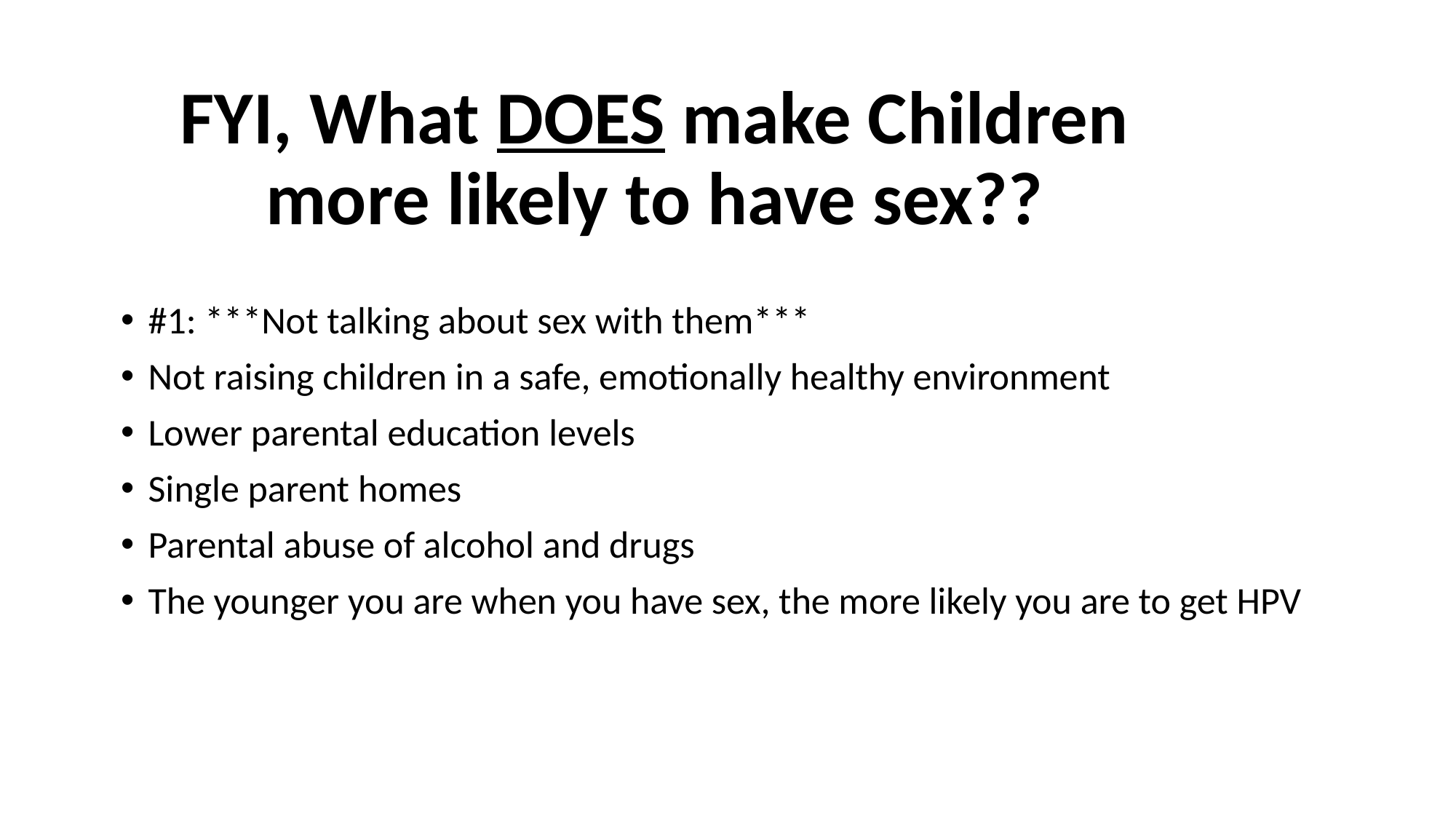

# FYI, What DOES make Children more likely to have sex??
#1: ***Not talking about sex with them***
Not raising children in a safe, emotionally healthy environment
Lower parental education levels
Single parent homes
Parental abuse of alcohol and drugs
The younger you are when you have sex, the more likely you are to get HPV

## Slide 10
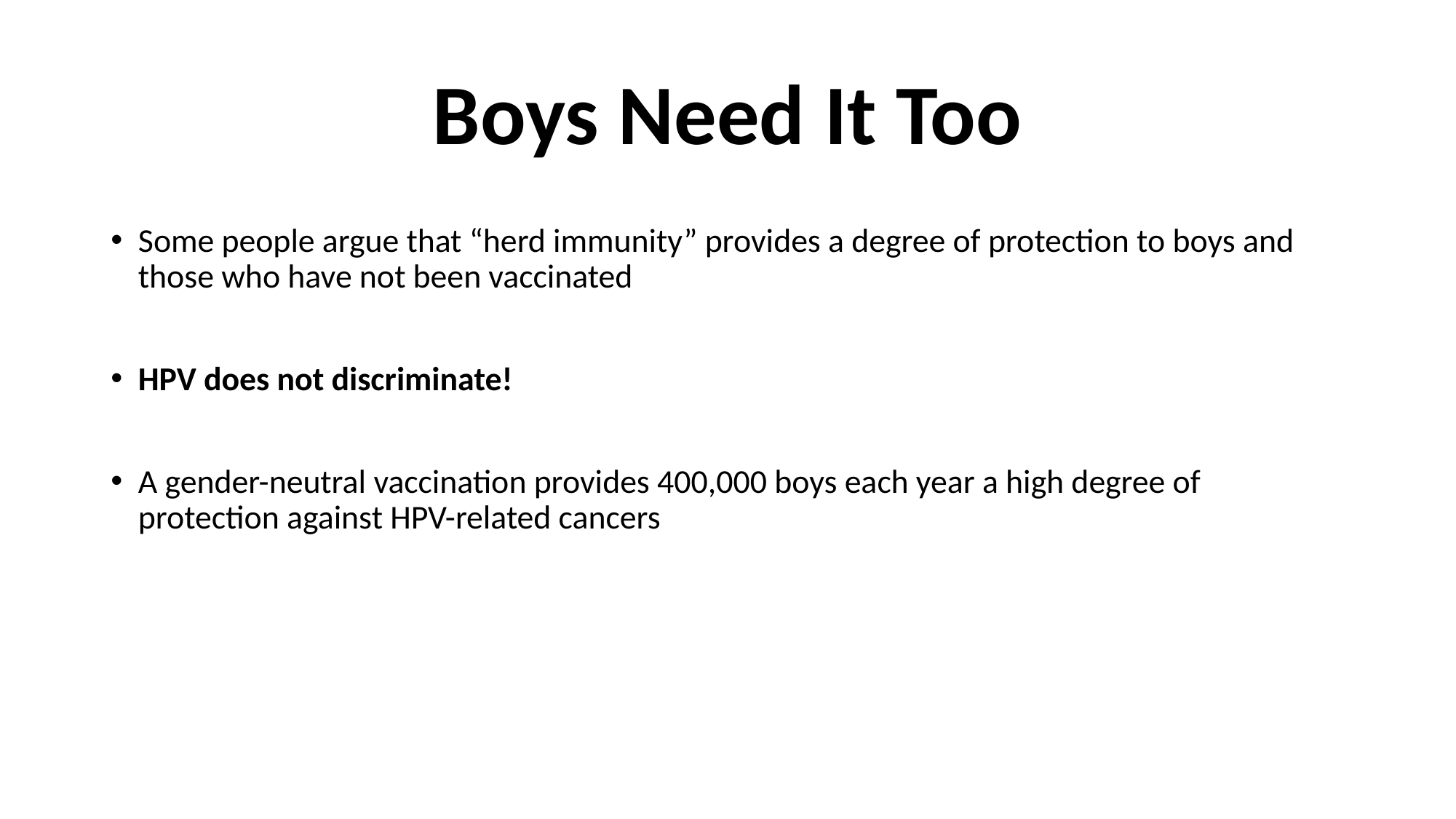

# Boys Need It Too
Some people argue that “herd immunity” provides a degree of protection to boys and those who have not been vaccinated
HPV does not discriminate!
A gender-neutral vaccination provides 400,000 boys each year a high degree of protection against HPV-related cancers

## Slide 11
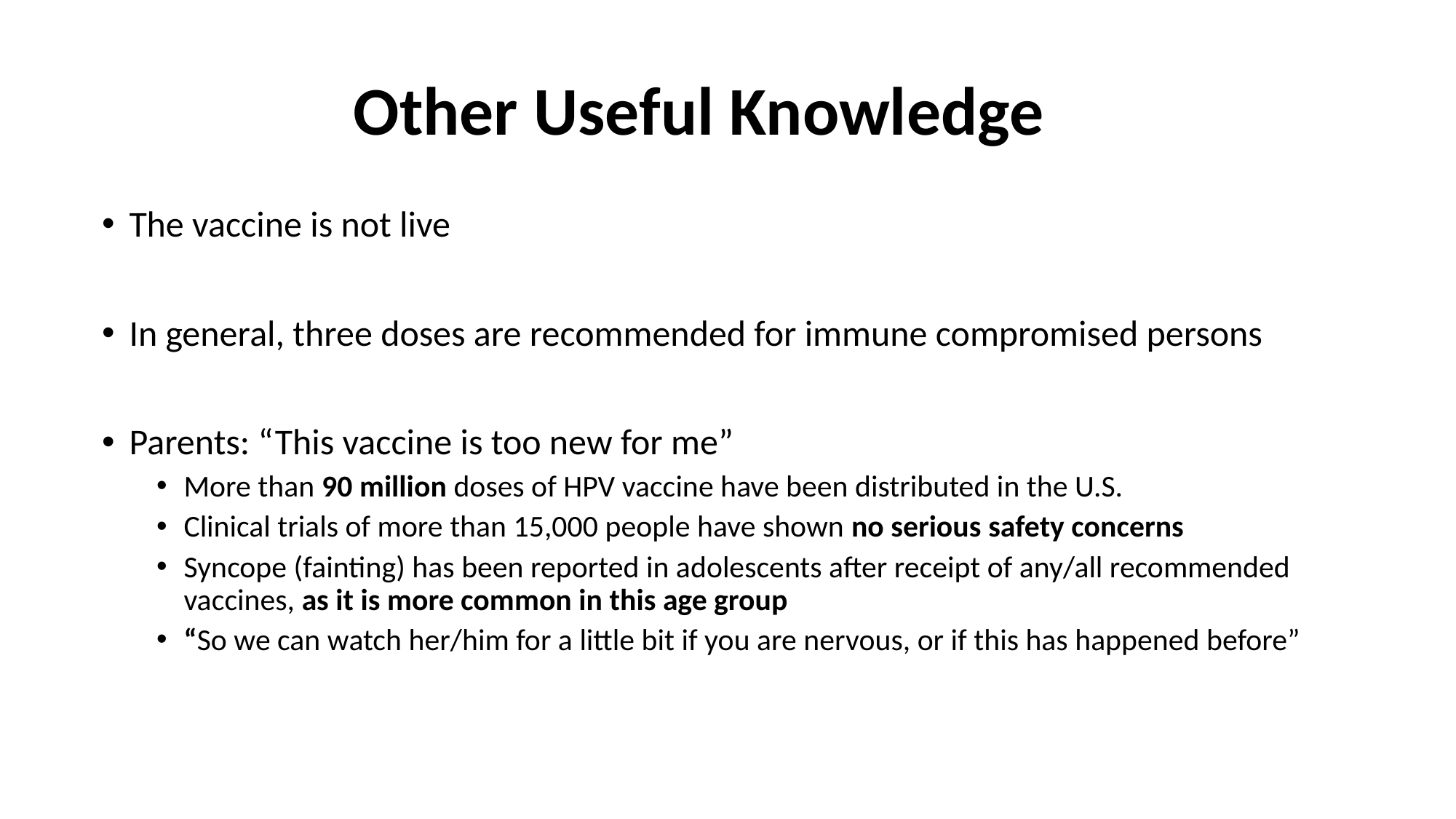

# Other Useful Knowledge
The vaccine is not live
In general, three doses are recommended for immune compromised persons
Parents: “This vaccine is too new for me”
More than 90 million doses of HPV vaccine have been distributed in the U.S.
Clinical trials of more than 15,000 people have shown no serious safety concerns
Syncope (fainting) has been reported in adolescents after receipt of any/all recommended vaccines, as it is more common in this age group
“So we can watch her/him for a little bit if you are nervous, or if this has happened before”

## Slide 12
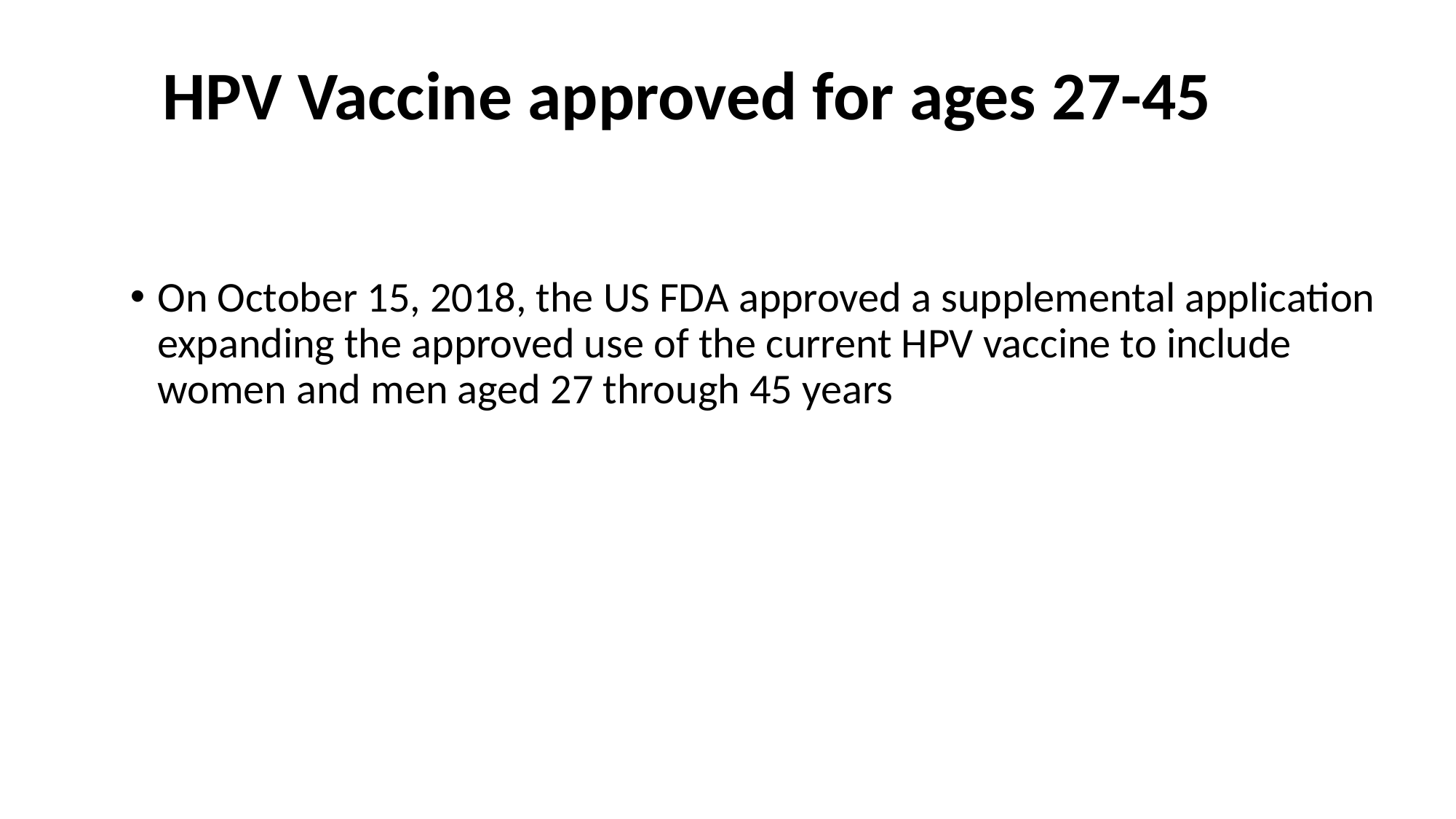

# HPV Vaccine approved for ages 27-45
On October 15, 2018, the US FDA approved a supplemental application expanding the approved use of the current HPV vaccine to include women and men aged 27 through 45 years

## Slide 13
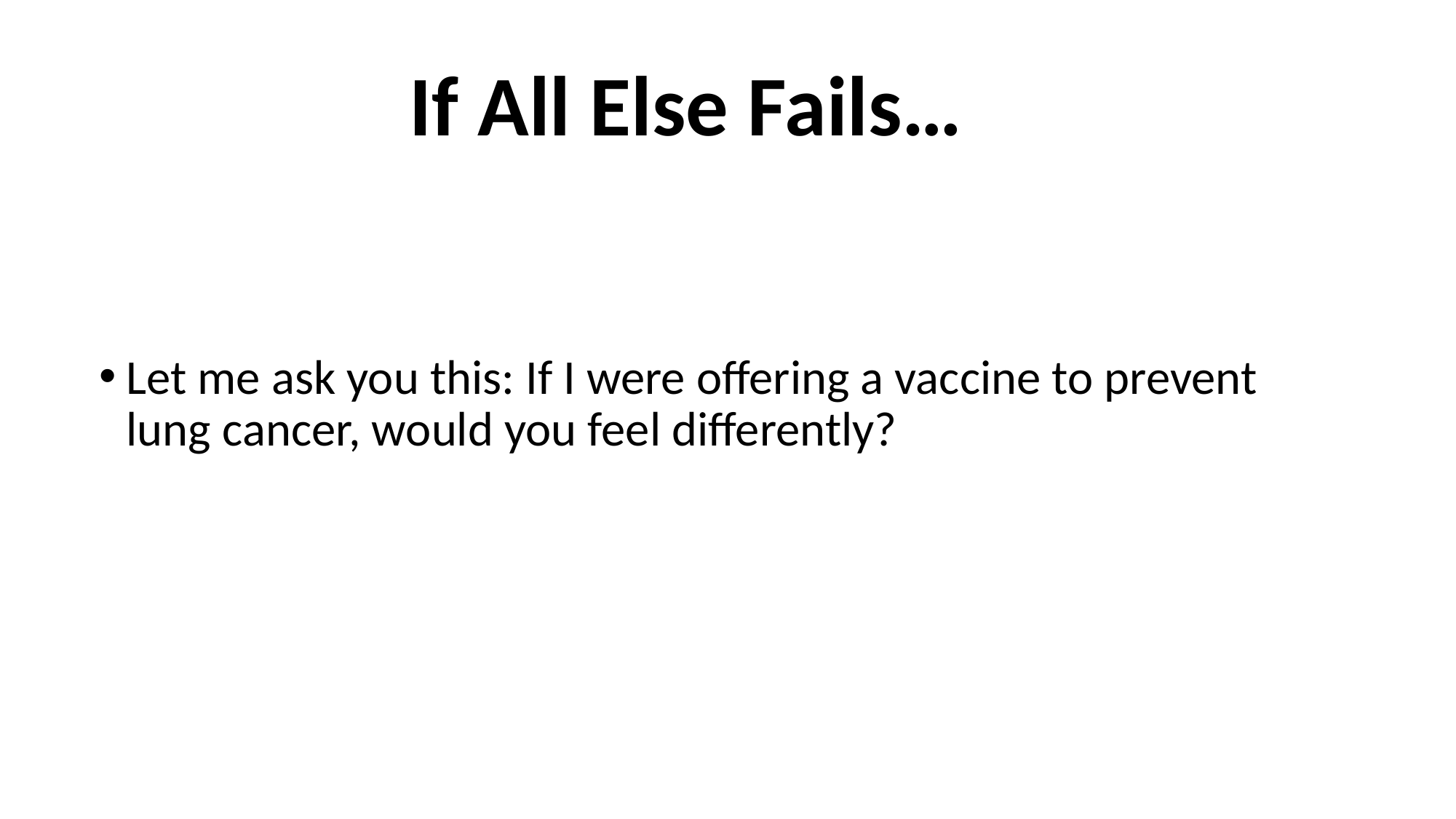

# If All Else Fails…
Let me ask you this: If I were offering a vaccine to prevent lung cancer, would you feel differently?

## Slide 14
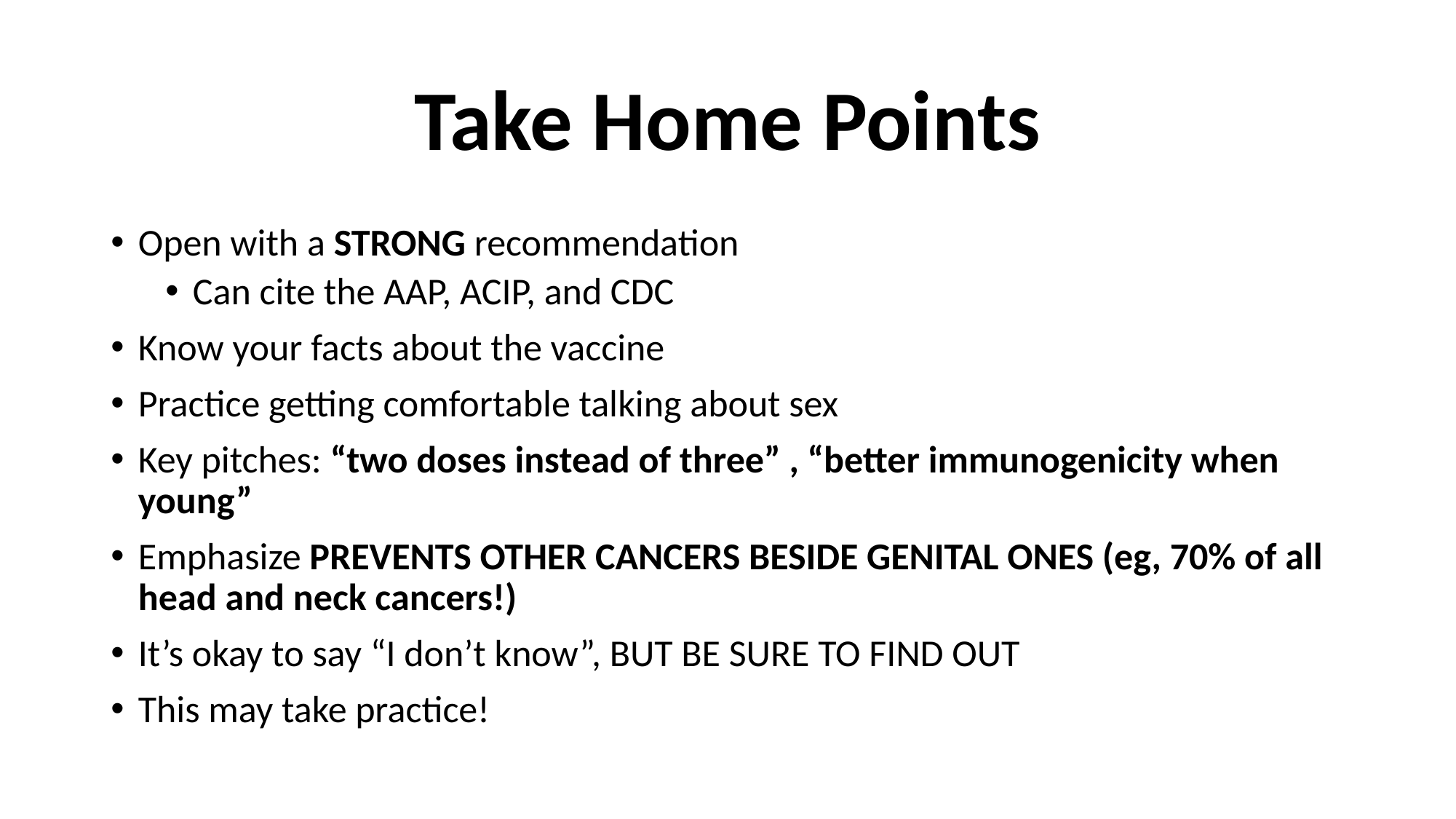

# Take Home Points
Open with a STRONG recommendation
Can cite the AAP, ACIP, and CDC
Know your facts about the vaccine
Practice getting comfortable talking about sex
Key pitches: “two doses instead of three” , “better immunogenicity when young”
Emphasize PREVENTS OTHER CANCERS BESIDE GENITAL ONES (eg, 70% of all head and neck cancers!)
It’s okay to say “I don’t know”, BUT BE SURE TO FIND OUT
This may take practice!

## Slide 15
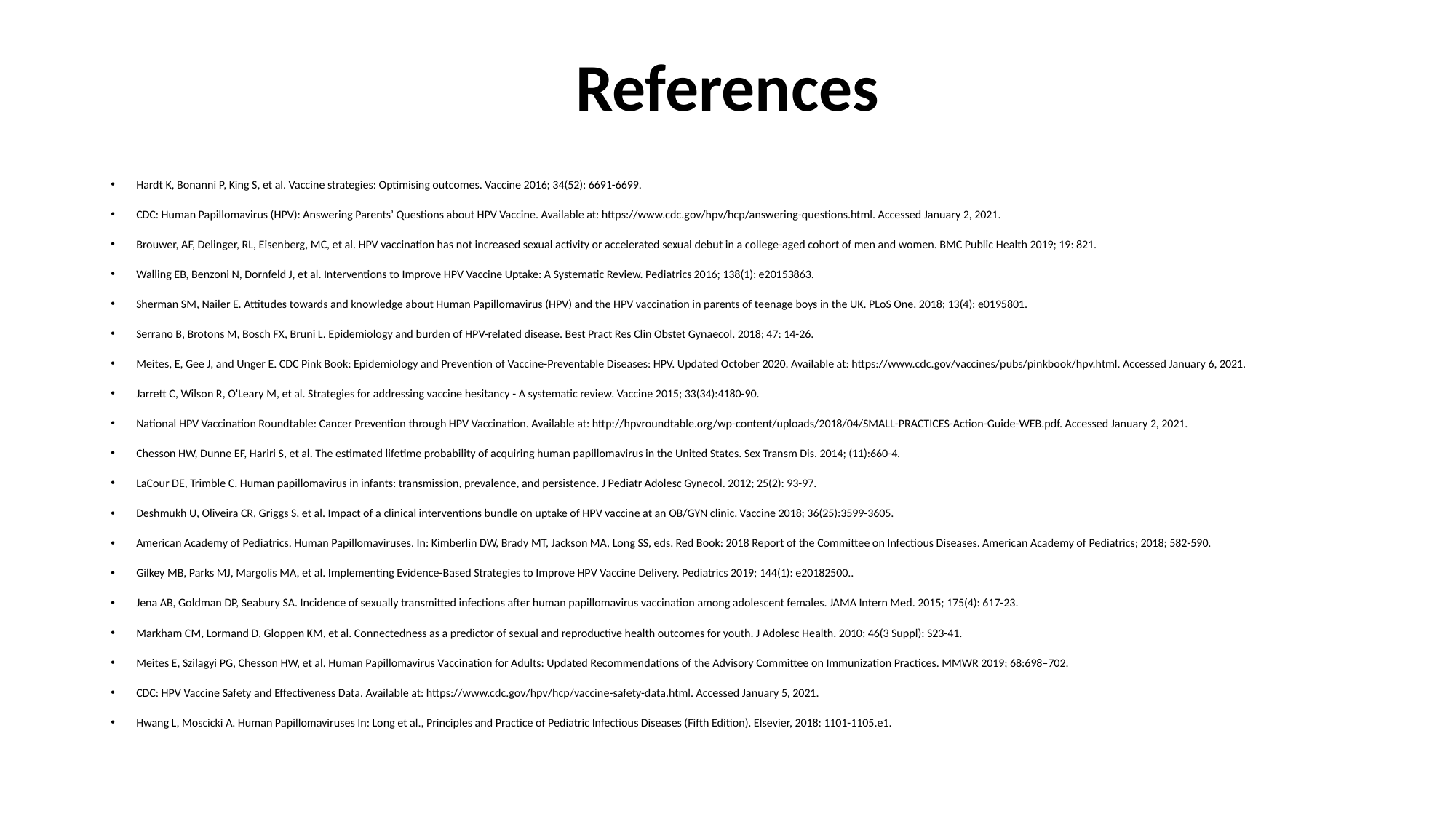

# References
Hardt K, Bonanni P, King S, et al. Vaccine strategies: Optimising outcomes. Vaccine 2016; 34(52): 6691-6699.
CDC: Human Papillomavirus (HPV): Answering Parents’ Questions about HPV Vaccine. Available at: https://www.cdc.gov/hpv/hcp/answering-questions.html. Accessed January 2, 2021.
Brouwer, AF, Delinger, RL, Eisenberg, MC, et al. HPV vaccination has not increased sexual activity or accelerated sexual debut in a college-aged cohort of men and women. BMC Public Health 2019; 19: 821.
Walling EB, Benzoni N, Dornfeld J, et al. Interventions to Improve HPV Vaccine Uptake: A Systematic Review. Pediatrics 2016; 138(1): e20153863.
Sherman SM, Nailer E. Attitudes towards and knowledge about Human Papillomavirus (HPV) and the HPV vaccination in parents of teenage boys in the UK. PLoS One. 2018; 13(4): e0195801.
Serrano B, Brotons M, Bosch FX, Bruni L. Epidemiology and burden of HPV-related disease. Best Pract Res Clin Obstet Gynaecol. 2018; 47: 14-26.
Meites, E, Gee J, and Unger E. CDC Pink Book: Epidemiology and Prevention of Vaccine-Preventable Diseases: HPV. Updated October 2020. Available at: https://www.cdc.gov/vaccines/pubs/pinkbook/hpv.html. Accessed January 6, 2021.
Jarrett C, Wilson R, O'Leary M, et al. Strategies for addressing vaccine hesitancy - A systematic review. Vaccine 2015; 33(34):4180-90.
National HPV Vaccination Roundtable: Cancer Prevention through HPV Vaccination. Available at: http://hpvroundtable.org/wp-content/uploads/2018/04/SMALL-PRACTICES-Action-Guide-WEB.pdf. Accessed January 2, 2021.
Chesson HW, Dunne EF, Hariri S, et al. The estimated lifetime probability of acquiring human papillomavirus in the United States. Sex Transm Dis. 2014; (11):660-4.
LaCour DE, Trimble C. Human papillomavirus in infants: transmission, prevalence, and persistence. J Pediatr Adolesc Gynecol. 2012; 25(2): 93-97.
Deshmukh U, Oliveira CR, Griggs S, et al. Impact of a clinical interventions bundle on uptake of HPV vaccine at an OB/GYN clinic. Vaccine 2018; 36(25):3599-3605.
American Academy of Pediatrics. Human Papillomaviruses. In: Kimberlin DW, Brady MT, Jackson MA, Long SS, eds. Red Book: 2018 Report of the Committee on Infectious Diseases. American Academy of Pediatrics; 2018; 582-590.
Gilkey MB, Parks MJ, Margolis MA, et al. Implementing Evidence-Based Strategies to Improve HPV Vaccine Delivery. Pediatrics 2019; 144(1): e20182500..
Jena AB, Goldman DP, Seabury SA. Incidence of sexually transmitted infections after human papillomavirus vaccination among adolescent females. JAMA Intern Med. 2015; 175(4): 617-23.
Markham CM, Lormand D, Gloppen KM, et al. Connectedness as a predictor of sexual and reproductive health outcomes for youth. J Adolesc Health. 2010; 46(3 Suppl): S23-41.
Meites E, Szilagyi PG, Chesson HW, et al. Human Papillomavirus Vaccination for Adults: Updated Recommendations of the Advisory Committee on Immunization Practices. MMWR 2019; 68:698–702.
CDC: HPV Vaccine Safety and Effectiveness Data. Available at: https://www.cdc.gov/hpv/hcp/vaccine-safety-data.html. Accessed January 5, 2021.
Hwang L, Moscicki A. Human Papillomaviruses In: Long et al., Principles and Practice of Pediatric Infectious Diseases (Fifth Edition). Elsevier, 2018: 1101-1105.e1.
