# Supplementary material for: Human Papillomavirus Knowledge and Communication Skills: A Role-Play Activity for Providers
Source: MedEdPORTAL. 2021 Apr 23;17:11150. doi: 10.15766/mep_2374-8265.11150 (PMC8063629; doi:10.15766/mep_2374-8265.11150)
Supplement: Supplementary file 1 — Facilitator Instructions.docxPre- and Postworkshop Self-Assessment.docxRole-Play Script.docxHPV Didactic Lecture.pptxSelf-Assessment Answer Key.docxRole-Play Rubric.docxPostparticipation Evaluation.docx [file mep_2374-8265.11150-s001.zip › E. Self-Assessment Answer Key.docx]

**Self-Assessment Answer Key**

1. Prior to the introduction of the HPV vaccine, what was the annual incidence of HPV in 15-24 year olds?
   1. **7 million infections/year**
   2. 1 million infections/year
   3. 500,000 infections/year
   4. 100,000 infections/year
2. What is the lifetime risk of acquiring HPV infection?
   1. **80%**
   2. 50%
   3. 25%
   4. 10%
3. Which of the following serotypes is not included in the Gardasil-9 HPV vaccine? (Not a high-risk serotype)
   1. 16
   2. 18
   3. 11
   4. **15**
4. What type(s) of cancer does the HPV vaccine prevent?
   1. Cervical cancer
   2. Penile cancer
   3. Oropharyngeal cancer
   4. A + B
   5. **All of the above**
5. Why is the HPV vaccine preferentially given at ages 9-12?
   1. It is before they are likely to engage in sexual activity
   2. There is a stronger immunogenicity of the vaccine in this age group
   3. Children require other vaccines around this age, and it is effective to “bundle” the vaccines together.
   4. **All of the above**
6. On a scale of 1-5 (1 being not at all comfortable, 5 being extremely comfortable), how comfortable are you talking about sex with parents?

○ ○ ○ ○ ○

Not at all Slightly Moderately Quite Extremely

comfortable comfortable comfortable comfortable comfortable

1. On a scale of 1-5 (1 being not at all confident, 5 being extremely confident), how confident are you in your ability to promote the HPV vaccine when talking to parents?

○ ○ ○ ○ ○

Not at all Slightly Moderately Quite Extremely

comfortable comfortable comfortable comfortable comfortable
