# Supplementary material for: Human Papillomavirus Knowledge and Communication Skills: A Role-Play Activity for Providers
Source: MedEdPORTAL. 2021 Apr 23;17:11150. doi: 10.15766/mep_2374-8265.11150 (PMC8063629; doi:10.15766/mep_2374-8265.11150)
Supplement: Supplementary file 1 — Facilitator Instructions.docxPre- and Postworkshop Self-Assessment.docxRole-Play Script.docxHPV Didactic Lecture.pptxSelf-Assessment Answer Key.docxRole-Play Rubric.docxPostparticipation Evaluation.docx [file mep_2374-8265.11150-s001.zip › F. Role-Play Rubric.docx]

**Role Play Script: Evaluation of Skills Rubric – to give to “Parent”**

Scenario: **Parent** is at **provider**’s office with her 11-year old daughter for a well-check.

Parent to check off points addressed below.

1. **Parent**: “Hello. I am here with my 11-year old daughter for a well-check appointment. I would like to know what vaccines are due for my child today?” (Prompt provider to give their “best pitch” of the HPV vaccine.)

□ Made a “strong recommendation”

□ Cited the CDC, AAP, and/or ACIP

□ Emphasized diseases caused by HPV besides cervical cancer (vaginal, vulvar, penile, anal, and/or oropharyngeal cancer; warts, respiratory papillomatosis)

□ Mentioned oropharyngeal cancer

□ “Bundled” vaccines together (eg., “your child is due for the tetanus booster, meningococcal vaccine, and HPV vaccine today.”)

□ Personalized approach (eg., “I made sure to get my child vaccinated for HPV, I recommend we give your daughter this vaccine as well.”)

1. **Parent**: “How do you get this disease?”

□ Mentioned sexual contact

□ Mentioned casual contact (eg., skin trauma, autoinoculation) or vertical transmission

(mother to child)

□ Mentioned fomite reservoirs (higher risk of plantar warts walking barefoot in public

showers)

1. **Parent**: “What are the chances she’ll get this disease in her lifetime?”

□ Correctly answered (80% risk)

1. **Parent**: “My daughter is not having sex yet! I’ll wait until she is older.”

□ Recommended to give at this age because it is prior to sexual activity

□ Emphasized “will only need two doses at this age”

□ Emphasized “immune response better among this age group”

**NOW SWITCH ROLES!!!**

1. **Parent**: “This vaccine is only going to encourage my daughter to have sex!”

□ Answered correctly (This is not true)

□ Cited factors that do encourage sex (not talking about sex with your children, low socioeconomic status, additional high-risk taking behaviors, etc.)

□ Cited the literature (eg., studies show us this is not true)/ offered to show parent the literature

1. **Parent**: “I’m sorry, but this vaccine really just seems too new for me.”

□ Reassured that it is not new (current vaccine came out in 2006)

□ Cite studies of >15,000 people in clinical trials showing no serious safety concerns or more than 90 million doses have been distributed in the U.S.

1. **Parent**: “Well, what are the side effects of this vaccine?”

□ Pain at the injection site mentioned

□ Two or more side effects mentioned (injection site pain/swelling/redness, fatigue, headache, fever, nausea, dizziness, malaise)

□ Syncope in age group mentioned

1. **Parent**: “This sounds good. Can I get this vaccine for myself, too??” (Note: Parent is 35 years old.)

□ Answered correctly (yes- you may receive this vaccine up to age 45.)

**Parent may award provider one bonus point each for citing any HPV-related statistics—examples: (can also refer to Powerpoint)**

□ Incidence of HPV in the pre-vaccine era

□ Prevalence of HPV in the pre-vaccine era

□ Incidence of cervical cancer in the US

□ Mortality of cervical cancer in the US

□ Incidence of HPV in 15-24 year olds in the pre-vaccine era
