# Supplementary material for: Human Papillomavirus Knowledge and Communication Skills: A Role-Play Activity for Providers
Source: MedEdPORTAL. 2021 Apr 23;17:11150. doi: 10.15766/mep_2374-8265.11150 (PMC8063629; doi:10.15766/mep_2374-8265.11150)
Supplement: Supplementary file 1 — Facilitator Instructions.docxPre- and Postworkshop Self-Assessment.docxRole-Play Script.docxHPV Didactic Lecture.pptxSelf-Assessment Answer Key.docxRole-Play Rubric.docxPostparticipation Evaluation.docx [file mep_2374-8265.11150-s001.zip › G. Postparticipation Evaluation.docx]

**Post-participation Evaluation**

1. The information presented in this workshop was:

a) Extremely useful

b) Somewhat useful

c) A little useful

d) Not at all useful

2. What percentage of the content in the workshop was new to you?

a) 0%

b) 25%

c) 50%

d) 75%

e) 100%

3. The length of this workshop was :

a) Too long

b) Just right

c) Too short

4. What changes to your practices do you plan to make as a result of this workshop?

5. Please use the space below to list any suggestions that may improve this workshop for the future:
