# Supplementary material for: Human Papillomavirus Knowledge and Communication Skills: A Role-Play Activity for Providers
Source: MedEdPORTAL. 2021 Apr 23;17:11150. doi: 10.15766/mep_2374-8265.11150 (PMC8063629; doi:10.15766/mep_2374-8265.11150)
Supplement: Supplementary file 1 — Facilitator Instructions.docxPre- and Postworkshop Self-Assessment.docxRole-Play Script.docxHPV Didactic Lecture.pptxSelf-Assessment Answer Key.docxRole-Play Rubric.docxPostparticipation Evaluation.docx [file mep_2374-8265.11150-s001.zip › A. Facilitator Instructions.docx]

**Facilitator Instructions**

Venue: Any group of faculty, 140 minutes

Facilitators: 1 faculty

**Workshop Schedule:**

| **Activity** | **Time** |
| --- | --- |
| Introduction | 5 minutes |
| Distribute and complete pre-intervention knowledge and skills self-assessment (evaluate HPV knowledge and self-perceived skills) | 5-10 minutes |
| Assign participants into pairs, designate one as the “provider” and one as the “parent” | 5 minutes |
| Pre-didactic role-play (skills assessment) with first four questions | 15 minutes |
| Participants to switch roles of “provider” and “concerned parent,” and role-play with last four questions | 15 minutes |
| Debriefing | 10-15 minutes |
| Didactic Lecture (Powerpoint presentation) | 20 minutes |
| Assign participants back into pairs, designate one as the “provider” and one as the “concerned parent” | 5 minutes |
| Post-didactic role-play (skills assessment) with first four questions | 15 minutes |
| Participants to switch roles of “provider” and “concerned parent,” and role-play with last four questions | 15 minutes |
| Debriefing | 5-10 minutes |
| Distribute and complete post-intervention knowledge and skills self-assessment (re-evaluate HPV knowledge and self-perceived skills) | 5-10 minutes |
| Wrap up and answer any questions | 5 minutes |

Practical instructions:

1. Introduction:

Introduce the workshop as a fun exercise for learners to improve their HPV knowledge base. Participants will be at different levels of training, which is expected.

1. Distribute and complete pre-intervention knowledge and skills self-assessment:

Advise participants that this assessment is only to increase their awareness of potential knowledge gaps.

1. Assign participants into pairs for pre-didactic role-play:

Distribute role-play script among pairs. Clarify that role-play questions were written based on true scenarios and common barriers identified by providers in giving the HPV vaccine. While the participants have different levels of training, you do not need to pair them up by level. Have participants get in groups of two (you may have one group of three if there is an odd number, but timing above is for groups of two). One participant will be the “provider.” The other participant will play the “concerned parent”-- this person will get the role-play script.

1. Pre-didactic role-play:

Instruct groups to complete the first four questions only. Set a timer for 15 minutes.

Some groups may finish ahead of others. The “concerned parent” may take notes on the “provider’s” responses to questions if desired.

After 15 minutes, instruct groups to switch roles. The new “concerned parent” will get the role-play script and may take notes on the provider’s responses to questions if desired. Instruct groups to complete the remaining four questions. Set a timer for 15 minutes.

Note: the same role-play script will be used for both role-plays, so you do not need to collect the scripts upon completion of the pre-didactic role-play.

1. Didactic Lecture:

The slides take approximately 20-25 minutes. Clarify that content in the powerpoint is based on CDC and AAP RedBook guidance.

Allow participants to answer questions before showing slide content. Examples:

- Would anyone like to volunteer their pitch of the HPV vaccine?
- Ask them about statistics while going through them: incidence, prevalence of HPV
- Can anyone name some of the HPV strains that are found in the vaccine?
- Does getting the HPV vaccine encourage children to have sex?
- What are some side effects of this vaccine?

1. Debriefing

Start by asking questions geared towards the “provider” role. Note: you do not have to use all of these questions unless time permits:

a) What did you think went well?

b) Did you find the questions difficult?

c) Were you given enough time to answer each question?

d) Was your role as the provider clear to you, given the provided introduction?

e) What could be improved about this session regarding the role of the “provider?”

Then ask questions addressing the “parent” role:

a) What do you think went well?

b) Were you satisfied with the provider’s answers?

c) Was your role as the parent clear to you, given the provided introduction?

d) What could be improved about this session regarding the role of the “parent?”

1. Assign participants back into same pairs for post-didactic role-play:

Instruct participants that the person who initially played the “provider” first will now play the “concerned parent” first. This ensures that each group member has an opportunity to answer all of the questions in the role-play.

Instruct the participants that when playing the “provider,” they should attempt to incorporate any and all points learned during the didactic lecture.

1. Post-didactic role-play:

Instruct groups to complete the first four questions only. Set a timer for 15 minutes.

The “concerned parent” may take notes on the “provider’s” responses to questions if desired.

After 15 minutes, instruct groups to switch roles. The new “concerned parent” will get the role-play script and may take notes on the provider’s responses to questions if desired. Instruct groups to complete the remaining four questions. Set a timer for 15 minutes.

When the timer goes off, facilitator may collect the role-play scripts from each group.

1. Debriefing

This debrief will focus more on the provider role and will take less time to complete.

a) What did you think went well?

b) Did you find the questions easier?

c) What new strategies did you try?

d) What could be improved about this session regarding the role of the “provider?”

1. Distribute and complete post-intervention knowledge and skills self-assessment:

Advise participants that this assessment is the same as the first one, and hopefully an improvement in scores will be appreciated.

1. Wrap-up and questions

Answer any other questions that participants may have. Assure them they will not be graded, and ask for feedback on any other gaps they have identified.

Materials: Powerpoint presentation, role-play script (can supply one set per group; back-up copies available in case one participant takes notes on the script), pre- and post-workshop quizzes.
