# Supplementary material for: Human Papillomavirus Knowledge and Communication Skills: A Role-Play Activity for Providers
Source: MedEdPORTAL. 2021 Apr 23;17:11150. doi: 10.15766/mep_2374-8265.11150 (PMC8063629; doi:10.15766/mep_2374-8265.11150)
Supplement: Supplementary file 1 — Facilitator Instructions.docxPre- and Postworkshop Self-Assessment.docxRole-Play Script.docxHPV Didactic Lecture.pptxSelf-Assessment Answer Key.docxRole-Play Rubric.docxPostparticipation Evaluation.docx [file mep_2374-8265.11150-s001.zip › B. Pre- and Postworkshop Self-Assessment.docx]

**Pre-/Postworkshop Self-Assessment**

1. Prior to the introduction of the HPV vaccine, what was the annual incidence of HPV in 15-24 year olds?
   1. 7 million infections/year
   2. 1 million infections/year
   3. 500,000 infections/year
   4. 100,000 infections/year
2. What is the lifetime risk of acquiring HPV infection?
   1. 80%
   2. 50%
   3. 25%
   4. 10%
3. Which of the following serotypes is not included in the Gardasil-9 HPV vaccine? (Not a high-risk serotype)
   1. 16
   2. 18
   3. 11
   4. 15
4. What type(s) of cancer does the HPV vaccine prevent?
   1. Cervical cancer
   2. Penile cancer
   3. Oropharyngeal cancer
   4. A + B
   5. All of the above
5. Why is the HPV vaccine preferentially given at ages 9-12?
   1. It is before they are likely to engage in sexual activity
   2. There is a stronger immunogenicity of the vaccine in this age group
   3. Children require other vaccines around this age, and it is effective to “bundle” the vaccines together.
   4. All of the above
6. On a scale of 1-5 (1 being not at all comfortable, 5 being extremely comfortable), how comfortable are you talking about sexuality with parents?

○ ○ ○ ○ ○

Not at all Slightly Moderately Quite Extremely

comfortable comfortable comfortable comfortable comfortable

1. On a scale of 1-5 (1 being not at all confident, 5 being extremely confident), how confident are you in your ability to promote the HPV vaccine when talking to parents?

○ ○ ○ ○ ○

Not at all Slightly Moderately Quite Extremely

confident confident confident confident confident
